# Range Sensor-Based Efficient Obstacle Avoidance through Selective Decision-Making

**DOI:** 10.3390/s18041030

**Published:** 2018-03-29

**Authors:** Youngbo Shim, Gon-Woo Kim

**Affiliations:** 1 Mechanical Technology Research Center, Korea Advanced Institute of Science and Technology, Daejeon 34141, Korea; shim36145@gmail.com; 2 School of Electronics Engineering, Chungbuk National University, Chungbuk 28644, Korea

**Keywords:** mobile robot, navigation, obstacle avoidance, decision-making, motion planning, local path planning

## Abstract

In this paper, we address a collision avoidance method for mobile robots. Many conventional obstacle avoidance methods have been focused solely on avoiding obstacles. However, this can cause instability when passing through a narrow passage, and can also generate zig-zag motions. We define two strategies for obstacle avoidance, known as Entry mode and Bypass mode. Entry mode is a pattern for passing through the gap between obstacles, while Bypass mode is a pattern for making a detour around obstacles safely. With these two modes, we propose an efficient obstacle avoidance method based on the Expanded Guide Circle (EGC) method with selective decision-making. The simulation and experiment results show the validity of the proposed method.

## 1. Introduction

The field of logistics automation is one of the fastest growing fields in mobile robot applications. Due to the rapid expansion of the online shopping industry, a significant increase in the use of logistics robots is being observed in logistics centers. The demand for logistics robots capable of unmanned operation is also increasing in order to cope with labor shortage problems, and to improve the efficiency of distribution centers. Mobile robot systems for automation of logistics, such as Amazon’s Kiva systems, Locus Robotics’ Locus Bot, and Alibaba’s Geek+ have been developed. Mobile robots for logistics automation are responsible for locating goods according to the users’ requests and delivering them safely to the desired locations. Conventionally, mobile robots in logistics operate on pre-defined paths and in human-free environments. However, it is necessary to perform the desired operation without a collision with an operator or a work vehicle in the dynamic environment. Under these circumstances, safety is more important than optimality from the viewpoint of robot navigation.

The work environment in the logistics and manufacturing industry is dynamic, where humans and robots coexist. A mobile robot operating in such environment may frequently encounter static or dynamic obstacles that are unexpected. In this scenario, the robot should find a safe path for reaching its destination within a given time, but finding a safe path by itself in real time is a challenging task. Therefore, an efficient collision avoidance algorithm is required, that allows the robot to smoothly avoid obstacles and travel safely to the destination while ensuring a real-time performance.

The Potential Field Method (PFM) has been studied as an initial method to solve the obstacle avoidance problem [1]. It is a method for calculating the direction in which the robot moves through the combination of the repulsive force from the obstacle and the attraction force to the destination. In this method, the disadvantage is that the path has to be planned in advance offline, so it cannot respond appropriately to the environment where the unknown obstacle exists. The Vector Field Histogram (VFH) generates polar histograms at regular intervals from sensor data [2]. In the algorithm, the final driving direction is selected by evaluating the most suitable sector from among all polar histogram sectors. The biggest problem with this method is that it does not take into account non-holonomic constraints. Similarly, the Dynamic Window Approach (DWA) converts the environment information expressed in the Cartesian coordinate system into the velocity space [3]. The approach is composed of the linear and angular velocity (v,w) axis and obtains an optimal obstacle avoidance control input considering the dynamics. Most of the above methods assume an omnidirectional type of robot and perform obstacle avoidance. If these methods are applied to differential-type or car-like robots, the user can suffer large performance degradation as compared to omni-directional robots [4,5]. Some methods have considered these types but fail to address the inherent problem of zig-zag motion and the issue of passing through narrow passages.

In order to overcome the above problems and to improve the degraded performance, VFH-based methods such as VFH+, VFH*, VPH(Vector Polar Histogram), and VPH+ have been studied. These methods interpret the environment information more efficiently and define an appropriate cost function [6,7,8,9]. The VFH method neglects the dynamics and the kinematics of a mobile robot like a differential drive robot. In addition, a problem of collision with an obstacle sometimes occurs without considering the volume of the robot. VFH+ considers the kinematics by approximating the trajectory of a mobile robot and takes into account the width of the mobile robot by using an implicit configuration space approach [6]. The VFH* combines with the A* algorithm for sensor-based obstacle avoidance to better deal with issues such as local minima and performance [7]. Based on the PFM and VFH+, the VPH was also proposed [8]. Unlike the VFH+, the VPH uses a polar histogram directly instead of the certainty grids of the Vector Field Histogram. This improvement contributes to an obvious reduction of computation and makes it suitable for obstacle avoidance. VPH+ mainly modifies by grouping objects into blocks, coordinating threaded caps, etc. As a result, it is more desirable than the VPH and VFH+ [9]. The DWA method has also been widely used since high-speed navigation can be obtained [10]. The improved DWA method, known as I-DWA, has been proposed, and applied to computing in real-time autonomous robot navigation [11]. In [11], an objective function involving the Lyapunov stability criterion has been suggested to ensure global and asymptotic convergence with the target while avoiding obstacles. Many of these methods have attempted to improve the navigation performance, and many attempts have been made to solve the inherent problems. However, instability can still occur when passing through a narrow passage, and a zig-zag motion can also be generated. The Follow the Gap Method (FGM) is an efficient algorithm to find the safest and closest gap among the observable gaps, solving the issues of passing through a narrow passage and zig-zag motion, as well as kinematic constraint problems [12]. This method has been proposed to find the gap between obstacles and to move the robot to the safest gap between them.

In order to cope with a sudden situation, input commands for collision avoidance should be generated in real time, based on information about the surrounding environment such as map and sensor data [13,14]. Conventional obstacle avoidance methods have been proposed to avoid obstacles in such a way that bypasses and maintains a certain distance from the closest and most interesting obstacles based on sensor data [1,2,10]. These methods generally show good performance, but often show incomplete performance in terms of navigation in specific environments such as doors, narrow corridors, etc. The FGM ensures efficient and stable passage through a narrow space between any obstacles. The major drawback is that of finding the gap even if a gap is not available. There is also an issue of trying to pass through the gap between obstacles, instead of finding a possible detour around obstacles. Therefore, to avoid obstacles in various environments, a complementary fusion of two methods needs to be considered, such as bypassing and passing through obstacles.

In this paper, we propose an efficient obstacle avoidance method called the improved Expanded Guide Circle (IEGC). The EGC method was originally proposed for the convenience of operation when operating the mobile robot remotely [15], with modification in the operational input for avoiding obstacles. Because of the EGC’s simplicity and proper performance, it has been applied to autonomous mobile robots. It is an algorithm that has a tendency to reach the destination through bypassing obstacles and does not have two driving patterns. It has found to have disadvantages such as zig-zag motion and a driving pattern focused on making a detour. Therefore, we have improved the EGC to more effectively avoid obstacles, and propose a selective decision-making process to determine whether to bypass or pass through obstacles. Unlike the previous methods, the proposed method has the advantages of having both driving patterns through the improved EGC and the selective decision-making process. The simulation and experiment results show the validity of the proposed method.

## 2. Overview of Approach

### 2.1. Definition of Two Types of Strategies for Obstacle Avoidance

The strategies for obstacle avoidance can be classified into two modes, the Entry mode and the Bypass mode. The Entry mode means that the robot passes between obstacles to reach its destination. For example, in Entry mode, a robot navigating in a hallway should pass through a door in order to enter a room without detouring around the door. In Bypass mode the robot bypasses towards space without obstacles in an environment with multiple obstacles.

**Figure 1:**
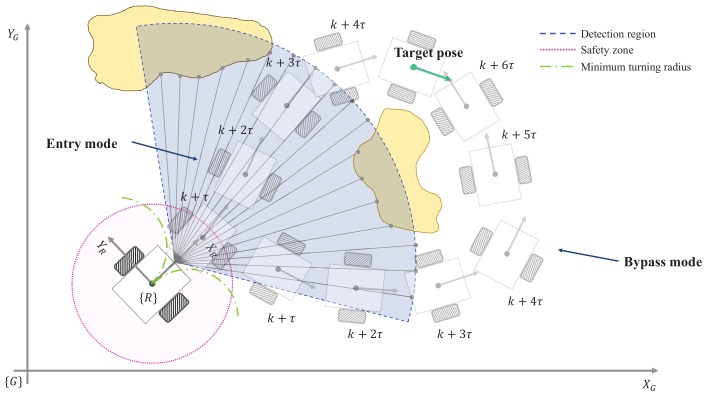
Definition of two driving modes with one target pose.

Most obstacle avoidance methods have bypass driving patterns. In the conventional method, zig-zag motion can occur in a narrow path due to the obstacle avoidance process. Even the robot sometimes fails to pass through the narrow passage. Also, it is nearly impossible to determine whether it is more efficient to pass through or to detour around obstacles using conventional methods. Recently, the FGM has been proposed as a method for avoiding obstacles by entering between them. The FGM defines a gap between obstacles and selects the gap that is considered as the safest among the gaps. The final driving direction is set through the sum of weights between the center direction of the gap that is considered to be the safest among the obstacles and directed towards the destination. This method guarantees the safest driving because it passes through the center between the obstacles when there is no local minimum. However, in many cases, it is more efficient to bypass the obstacles, not to pass through dense obstacles, as shown in Figure 1, in time. When detouring around the obstacles, the safety of robots can be assured more reliably.

It is difficult to solve the fundamental problem of avoiding obstacles if we try to solve it using only one of the two methods. Therefore, in order to avoid obstacles effectively, it is necessary to use the combined of both the Entry and Bypass modes. Hence, we propose this effective obstacle avoidance method considering both driving patterns from the next section.

### 2.2. Kinematics of Wheeled Mobile Robots

**Figure 2:**
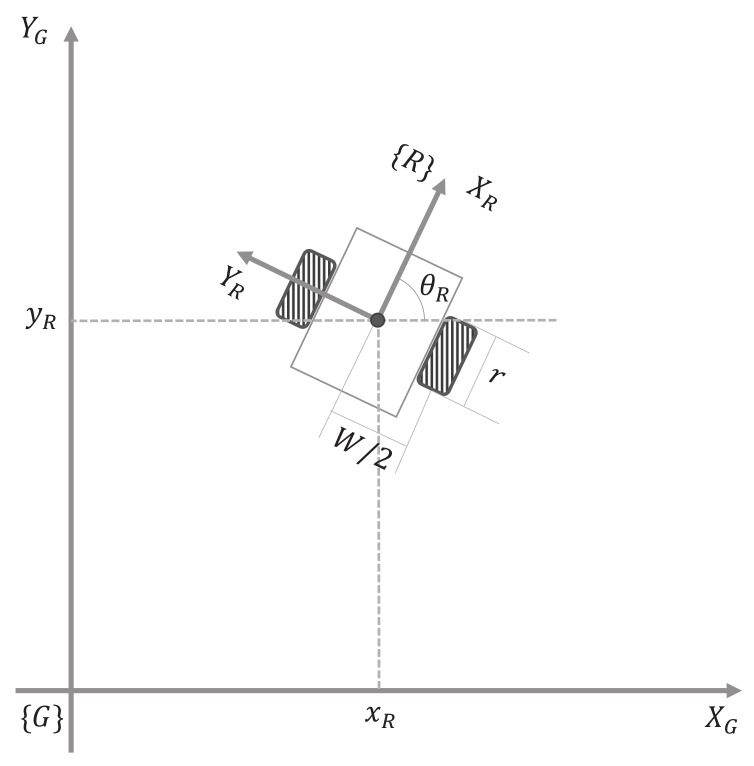
Definition of the reference frames: global reference frame {G} and robot frame {R}.

For the derivation from the kinematic mode of wheeled mobile robots, we define the dimensionality of a robot on the plane as three—two for position in the plane and one for orientation, which is in contrast to the global reference frame and the robot frame (shown in Figure 2). The pose of a mobile robot in the global frame can be represented as:

(1) $$q_{R}={\left[x_{R}\;y_{R}\;\theta_{R}\right]}^{T}$$

where $x_{R}$, $y_{R}$, and $\theta_{R}$ denote the position in the global reference frame and orientation of a mobile robot, respectively. The kinematic model of a differential drive robot with a wheel radius *r* can be based on the following equation by using Jacobian matrix as:

(2) $$\begin{array}{cc} {\dot{q}}_{R} & =J_{kin}\cdot \dot{\phi }\\ & where\;J_{kin}=\left(\begin{array}{cc} r\cos\theta_{R}/2 & r\cos\theta_{R}/2\\ r\sin\theta_{R}/2 & r\sin\theta_{R}/2\\ r/W & -r/W \end{array}\right) \end{array}$$

where $\dot{\phi }={\left[{\dot{\phi }}_{r}\;\;{\dot{\phi }}_{l}\right]}^{T}$ is the rotating speed of each wheel, and *W* is the distance between two wheels. A mobile robot is controlled by the linear velocity and angular velocity, *v* and *w*, respectively. According to the kinematics, the relationship between the linear and angular velocity *v*, *w* and the rotating speed $\dot{\phi }$ is derived as:

(3) $$\begin{array}{c} v_{R}=\frac{{\dot{\phi }}_{l}+{\dot{\phi }}_{r}}{2},\;w_{R}=\frac{{\dot{\phi }}_{l}-{\dot{\phi }}_{r}}{W} \end{array}$$

If the pose of a mobile robot needs to be determined from the linear and angular velocity, it can be acquired as:

(4) $${\dot{q}}_{R}=T\left(q_{R}\right)\cdot \left(\begin{array}{c} v_{R}\\ w_{R} \end{array}\right)\;where\;T\left(q_{R}\right)=\left(\begin{array}{cc} \cos\theta_{R} & 0\\ \sin\theta_{R} & 0\\ 0 & 1 \end{array}\right)$$

## 3. Efficient Collision Avoidance Based on the Expanded Guide Circle Method With Selective Decision-Making

The Expanded Guide Circle (EGC) [15] is an efficient method to induce the robot to move toward a safer area according to the user’s command, in order to the increase convenience and safety of remote operation. The EGC method is a simple algorithm, and shows good performance compared with the existing obstacle avoidance algorithms [1,2,10]. However, since it is an algorithm that mainly considers an avoidance driving pattern as in the previous methods, it generates a control input that is not suitable for the driving situation of entry between two obstacles or a narrow passage. So, we propose the efficiently combined method to solve these issues based on an improved EGC method.

### 3.1. Defining the Initial Guide Circle (IGC)

If the mobile robot encounters some unexpected obstacles while traveling at a certain speed, it has to move towards the safest area among the movable areas or make a sudden brake operation. To ensure safety, the worst case should be considered. The worst safety threat situation for a robot is when there are obstacles in a braking zone within the minimum distance that the robot needs to stop completely. The braking section is the minimum safety zone of the robot and obstacles should not be allowed to enter the safety zone. Therefore, the minimum distance of the braking section can be defined as the minimum safe distance, and the area within this distance is defined as an unsafe region, as shown below.

(5) $$r_{ur}=\frac{v_{target}^{2}-v_{current}^{2}}{2\cdot a}$$

where $v_{target}$ is the desired velocity after the robot reduces its speed, $v_{current}$ is the current velocity, and *a* is the maximum acceleration. In the above equation, the acceleration has a negative value because it is formulated from the braking distance.

In the case of Ackerman steering and a differential-drive mobile robot with a non-holonomic constraint, robot motions are restricted by a minimum turning radius [16,17]. The differential-drive robot can be approximated by a fully holonomic robot by first turning to the desired direction and then driving straight to the target point. However, it is supposed to be non-holonomic for smooth and efficient driving. The mobile robot with Ackerman steering is car-shaped with a fixed front wheel with a center pivot point. Therefore, only the control input within the minimum turning radius is effective when such kinematics are involved. The interior of a circle with a radius $r_{ur}$ is defined as an unsafe region. The center of the Initial Guide Circle (IGC) is defined as a point at which a mobile robot meets the boundary of the unsafe region when traveling in the minimum turning radius.

(6) $$P_{LIGC}=\left(\begin{array}{c} x_{LIGC}\\ y_{LIGC} \end{array}\right)=\left(\begin{array}{c} r_{ur}\cdot \cos\left(-w_{max}/2\right)\\ r_{ur}\cdot \sin\left(-w_{max}/2\right) \end{array}\right)$$

(7) $$P_{RIGC}=\left(\begin{array}{c} x_{RIGC}\\ y_{RIGC} \end{array}\right)=\left(\begin{array}{c} r_{ur}\cdot \cos\left(w_{max}/2\right)\\ r_{ur}\cdot \sin\left(w_{max}/2\right) \end{array}\right)$$

**Figure 3:**
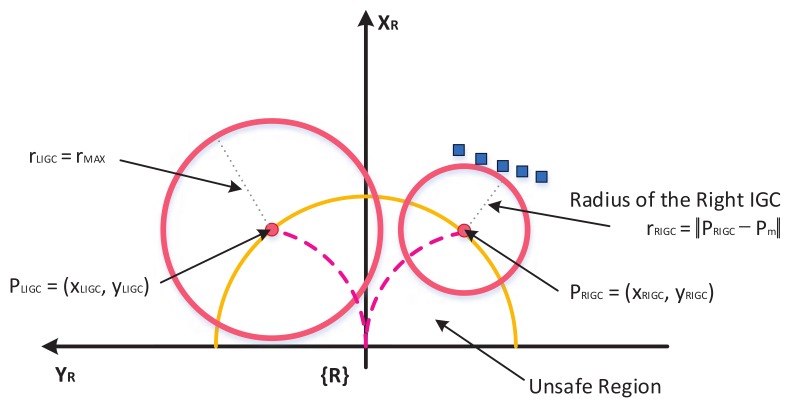
Defining the Initial Guide Circle (IGC) in the robot reference frame {R}.

Range sensors such as LiDAR(Light Detection and Ranging), sonar, etc. can be used to detect obstacles. In this paper, we developed the LiDAR sensor-based approach; the scan data set of LiDAR is $M=\{m_{i}:ith\;scan\;data\;on\;the\;point\;P_{m_{i}}=\left(x_{i},y_{i}\right),\;i=1,\ldots ,n\}$. The radius of the Guide Circle (GC) is the distance from the center of the GC to the nearest scan data and it represents the safety of the area around the center. If the distance exceeds $r_{max}$, it is determined as $r_{max}$.

(8) $$P_{m}^{LIGC}=\underset{P_{m_{i}}}{\mathrm{argmin}}∥P_{LIGC}-P_{m_{i}}∥,\;for\;m_{i}\in M,\;i=1,\ldots ,n$$

(9) $$r_{LIGC}=\left\{\begin{array}{cc} ∥P_{LIGC}-P_{m}^{LIGC}∥ & if\;∥P_{LIGC}-P_{m}^{LIGC}∥<r_{max}\\ \;r_{max} & otherwise \end{array}\right.$$

(10) $$P_{m}^{RIGC}=\underset{P_{m_{i}}}{\mathrm{argmin}}∥P_{RIGC}-P_{m_{i}}∥,\;for\;m_{i}\in M,\;i=1,\ldots ,n$$

(11) $$r_{RIGC}=\left\{\begin{array}{cc} ∥P_{RIGC}-P_{m}^{RIGC}∥ & if\;∥P_{RIGC}-P_{m}^{RIGC}∥<r_{max}\\ \;r_{max} & otherwise \end{array}\right.$$

where the formulas for finding the left initial GC and the right initial GC are similar. Depending on how $r_{max}$ is determined, it is decided whether to avoid obstacles far away or to avoid obstacles. Therefore, it is recommended to select $r_{max}$ by considering the volume of the robot and the appropriate distance. If the radius of the left and right GCs are both greater than $r_{max}$, no further processing is necessary and the control input of the robot is valid. Therefore, the mobile robot is controlled using the control input generated in the destination direction. On the contrary, if either the $r_{LIGC}$ and $r_{RIGC}$ is less than $r_{max}$, a control command for avoidance is generated, as shown in Figure 3.

### 3.2. Defining the Auxiliary Guide Circle (AGC)

In [15], the direction for avoiding is determined by the reference line because the GC should be expanded into the direction for avoiding the obstacle. The direction selection method for obstacle avoidance results in a zig-zag motion problem, and sometimes no intersection can be generated. Therefore, it is necessary to define the new Auxiliary Guide Circle (AGC) for analyzing and solving the fundamental problems of existing algorithms and improving performance.

**Figure 4:**
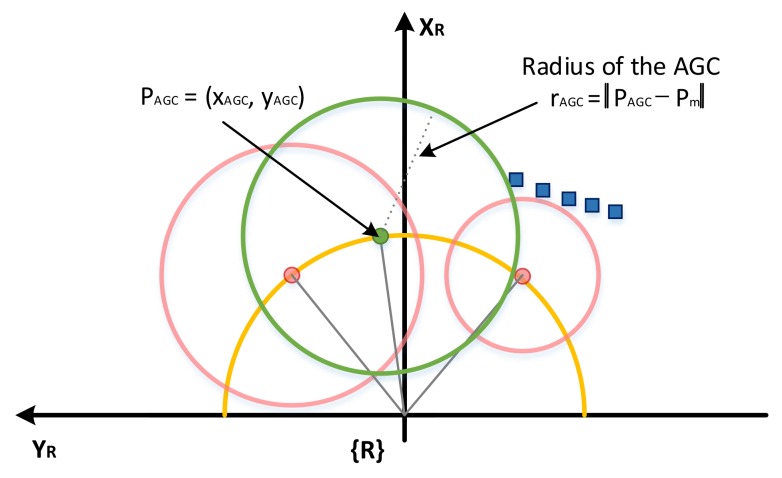
Defining the Auxiliary Guide Circle (AGC) in the robot reference frame {R}.

The center of every Guide Circle (GC) must always have a solution. Considering the kinematic constraints, all points between the center points of both the left and right IGCs can be converted into control inputs, as shown in Figure 4. The radius of the initial guide circle is used to calculate the safer control input among the feasible control inputs. Each radius represents the safety on the left and right sides, and the center of the AGC is calculated by the weighted sum of the angles of the center points in order to find the distance between the center of the auxiliary GC and the nearest scan data. The same method as calculated by IGC is defined as:

(12) $$\theta_{AGC}=\frac{r_{RIGC}\cdot \theta_{RIGC}+r_{LIGC}\cdot \theta_{LIGC}}{r_{RIGC}+r_{LIGC}}$$

(13) $$P_{AGC}={\left[r_{ur}\cdot cos\left(\theta_{AGC}\right)\;r_{ur}\cdot \sin\left(\theta_{AGC}\right)\right]}^{T}$$

(14) $$r_{AGC}=\left\{\begin{array}{cc} ∥P_{AGC}-P_{m}^{AGC}∥ & if\;∥P_{AGC}-P_{m}^{AGC}∥<r_{max}\\ r_{max} & otherwise \end{array}\right.$$

In Equation (12), $\theta_{RIGC}$ and $\theta_{LIGC}$ are calculated from $P_{RIGC}$ and $P_{LIGC}$, respectively, based on the $X_{R}$ axis in the robot frame {R}. As shown in Equation (13), the weighted angle is converted to a point and defined as the center of the AGC. The radius of the AGC is obtained in the same way as the radius of the initial Guide Circle.

### 3.3. Defining the Expanded Guide Circle (EGC) with Selective Decision-Making

Finally, there are two steps to define the EGC. The first step is to decide whether to enter between obstacles or bypass obstacles. The process of this step can be changed flexibly according to the characteristics of the robot used by the user. In this paper, we set the decision factor to reach the destination safely and quickly in the navigation field of the mobile robot. The process of deciding whether to enter or bypass this factor is called the “decision process”. The decision process not only decides whether to enter or not, but also finds a destination where the robot should approach. In the second step, the robot combines with the EGC to determine the direction in which the robot should travel. The above two steps are the most important contribution to this paper, considering both the driving pattern and the bypass driving pattern in order to avoid obstacles efficiently. These two steps are described in detail in the next section.

Selective decision-making, including the “decision process”, is the method for selectively determining the more efficient mode of driving: entering or bypassing. The sequence of the selective decision-making method is briefly as follows. First, calculate the spacing between all obstacles within the observable range, for example with the FGM. In addition, determine whether there is the best gap that can be entered through among the intervals calculated through the decision process, and determine the driving mode of entering and bypassing. If there is a gap to enter, the center point of the gap is determined as the goal, otherwise, the original destination is determined as the goal. The selective decision-making method proceeds in this sequence. The decision process is described in more detail below:

#### 3.3.1. Finding Feasible Gaps the Robot Can Pass Through

**Figure 5:**
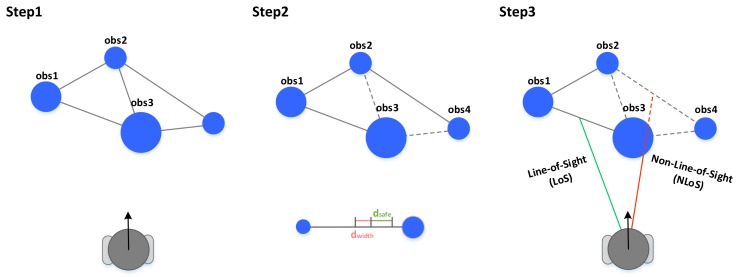
Sequence for finding safe gaps to enter.

As shown in Figure 5, after all the distances of observable gaps are calculated, all gaps are evaluated to determine if the robot can pass through safely, and it is necessary to determine whether the center of the gap is in the line-of-sight (LoS). If the goal is selected as the center of the gap that is not in the LoS, the robot should avoid the obstacle existing between the center of the gap and the robot, and go to the center of the gap. Therefore, the gap with the center point on non-LoS is excluded from being the best gap candidate because it can cause inefficient driving.

#### 3.3.2. Calculating the Cost of the Path and Deciding the Driving Pattern

**Figure 6:**
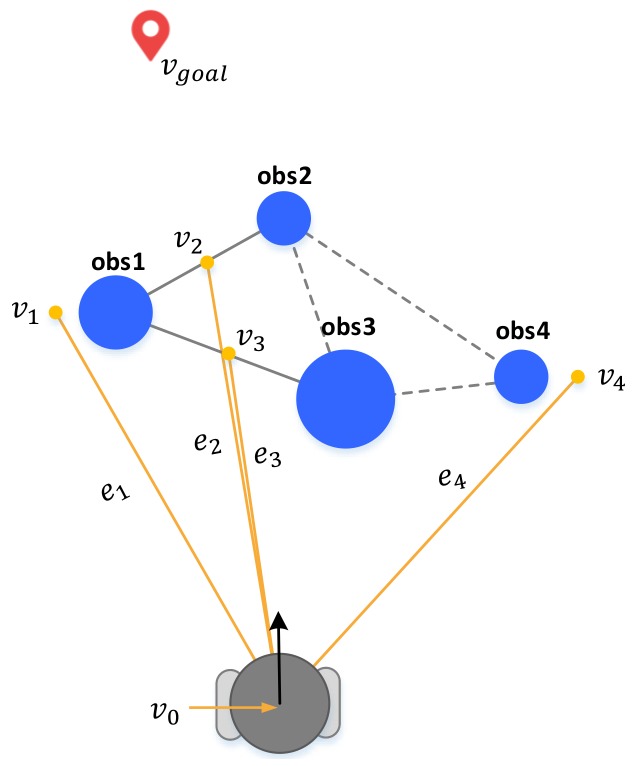
Method to find the feasible path for minimizing the path cost using the cost function.

In this step, the cost function is defined and the cost is used in order to select the most efficient path among the gaps selected in 3.3.1 and bypass the obstacles. Remember that cost function can be defined differently according to the user’s purpose and the characteristics of the robot. For example, in the case of robots carrying heavy loads, it is more efficient to move around complex obstacles for safety, but it is better to go between obstacles in the case of cleaning robots or exploratory robots. The cost function of a general mobile robot is defined as follows:

(15) $$P=\left\{P_{1},\;P_{2},\;P_{3},\ldots \right\},\;where\;P_{i}=\left\{v_{0},\;v_{i},\;v_{goal}\right\}$$

(16) $$J\left(P_{i}\right)=g\left(e_{i}\right)+h\left(v_{i},v_{goal}\right)$$

where *P* is a set of paths and each path consists of vertices which are represented as $v_{i}$. In Figure 6, paths $P_{1}$ and $P_{4}$ including $v_{1}$ and $v_{4}$ are paths that bypass obstacles. The others are paths with the vertex of the center of the passable gap. First, g(·) is the cost of the edge connecting the robot and the vertex, and h(·) is the heuristic cost between $v_{1}$ and $v_{goal}$, which is defined as the 2-norm of $v_{i}$ and $v_{goal}$ in this paper. Finally, the cost function for any path is defined as the sum of *g* and *h*, as in Equation (16). For the robot to reach its destination quickly, it needs to find the shortest path. This is the same problem as finding the path that minimizes the cost function.

(17) $$P^{*}=\underset{P_{i}\in P}{\mathrm{argmin}}\;J\left(P_{i}\right)$$

The best path found in Equation (17) has the lowest cost function among the elements of the set of paths. If the best path is the center of the gap, it has an entry driving pattern or a bypassing pattern. The output of the decision process is the vertex that is the element of the path, and the driving pattern also depends on the output. Therefore, the goal is switched to either the original destination or the output depending on the presence of the output.

(18) $$P_{goal}=\left\{\begin{array}{cc} P^{*} & if\;P^{*}\;is\;valid.\\ {\left[x_{goal}\;y_{goal}\right]}^{T} & otherwise \end{array}\right.$$

(19) $$\theta_{goal}=\tan^{-1}\left(\frac{P_{goal}\cdot y}{P_{goal}\cdot x}\right)$$

The IGC represents the radius of safety with respect to the left and right area in the direction the robot is going to travel through. If it travels in the center of the AGC, the mobile robot can move to a safe area locally. However, it is also important that the robot reaches its destination quickly and safely while avoiding obstacles. Therefore, we combine the center of the AGC to go to the safe area in the newly obtained destination direction.

(20) $$\begin{array}{cc} \theta_{IEGC} & =\frac{\left(r_{AGC}-r_{min}\right)}{\left(r_{AGC}-r_{min}\right)+\frac{\left(r_{max}-r_{min}\right)}{\left(r_{AGC}-r_{min}\right)}}\cdot \theta_{goal}\\ & +\frac{\frac{\left(r_{max}-r_{min}\right)}{\left(r_{AGC}-r_{min}\right)}}{\left(r_{AGC}-r_{min}\right)+\frac{\left(r_{max}-r_{min}\right)}{\left(r_{AGC}-r_{min}\right)}}\cdot \theta_{AGC} \end{array}$$

(21) $$P_{IEGC}={\left[r_{ur}\cdot \cos\left(\theta_{IEGC}\right)\;r_{ur}\cdot \sin\left(\theta_{IEGC}\right)\right]}^{T}$$

(22) $$r_{IEGC}=\left\{\begin{array}{cc} ∥P_{IEGC}-P_{m}^{IEGC}∥ & if\;∥P_{IEGC}-P_{m}^{IEGC}∥<r_{max}\\ \;r_{max} & otherwise \end{array}\right.$$

Equation (20) is a formula expressing $\theta_{IEGC}$, which is the final driving direction of the robot. It decides whether to travel towards the destination or focus more on avoiding obstacles, depending on the size of $r_{AGC}$. If the radius of the left and right GCs in the initial GC is larger than $r_{max}$, the robot does not need to proceed to the next step of the algorithm and can be controlled by the original control input. However, if it is smaller than $r_{max}$ and larger than $r_{min}$, the extension GC is calculated to avoid the obstacle as shown in Equation (20). The larger the $r_{AGC}$ is, the nearer it gets to the destination, and the smaller the $r_{AGC}$ is, the more it detours to avoid obstacles. Finally, if $r_{AGC}$ is smaller than $r_{min}$, there is a risk of colliding with the linear velocity of $\theta_{AGC}$, so it is safe to move only at the minimum linear velocity or angular velocity. The control command is calculated by the EGC as:

(23) $$v_{R}^{mod}=\left\{\begin{array}{cc} \;v_{input} & if\;r_{LIGC}\geq r_{max},\;r_{RIGC}\geq r_{max}\\ \left(r_{IEGC}/r_{min}\right)\cdot v_{input} & otherwise \end{array}\right.$$

(24) $$w_{R}^{mod}=\left\{\begin{array}{cc} \;w_{input} & if\;r_{LIGC}\geq r_{max},\;r_{RIGC}\geq r_{max}\\ 2\cdot \tan^{-1}\left(\frac{P_{IEGC}\cdot y}{P_{IEGC}\cdot x}\right) & otherwise \end{array}\right.$$

In Table 1, the improved EGC algorithm is described in pseudo code form. The experiments described in the next section are based on it. In the next section, we verify the performance of the proposed method through simulations and experiments.

## 4. Performance Evaluation using Simulation and Experiment Results

Before experimenting with the method proposed in the real world, we use Matlab to perform the simulation. The performance of the proposed method is compared with the Dynamic Window Approach (DWA) [3], and the EGC [15], proposed for the convenience of remote operation. We compare the robot’s trajectory and the time it takes to reach the destination with the environment configuration in which a single obstacle or multiple obstacles exist.

The parameter settings of the three methods shown in this experiment are as follows. First, the DWA has a parameter heading that causes the direction of the robot to converge with the destination, a parameter clearance which represents the distance to the obstacle, and a parameter velocity that deals with the magnitude of the velocity to reach the destination quickly. The performance of DWA is changed very sensitively depending on how these parameters are determined. Experimental parameters were modified to determine heading and clearance at 0.1 and velocity at 0.12.

The extension guide circle has a control cycle *T*, a constant *k* multiplied by the control cycle *T*, and $r_{min}$ and $r_{max}$ to be determined by the Safety Index Circle (SIC). The four parameters were determined to be 0.2 (s), 5, 0.3 (m), and 0.8 (m), respectively. In the proposed method, it is determined that the $r_{ur}$ values obtained from the robot width and braking distance are 0.5 (m) and 0.7 (m), respectively. $r_{min}$ and $r_{max}$ are 0.2 (m) and 1.0 (m), respectively.

The maximum linear velocity of all methods used in the experiment is 0.5 m/s and the maximum angular velocity is $\pm \frac{\pi }{4}$. Experimental results show that the algorithm works in the global coordinate system and the robot coordinate system, and in the control input results over time. It is verified that the proposed method is better in terms of performance than the other methods.

### 4.1. Simulation in the Environment with a Single Obstacle

**Figure 7:**
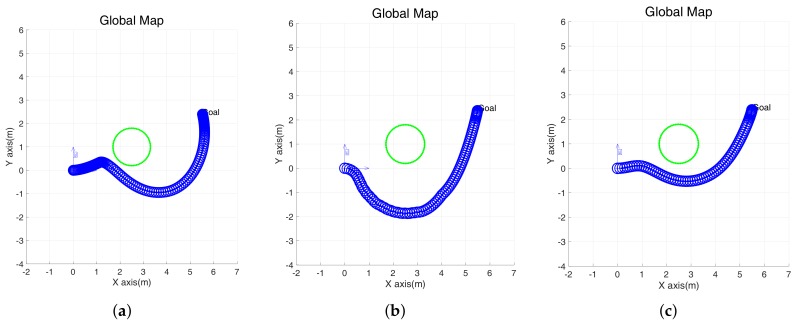

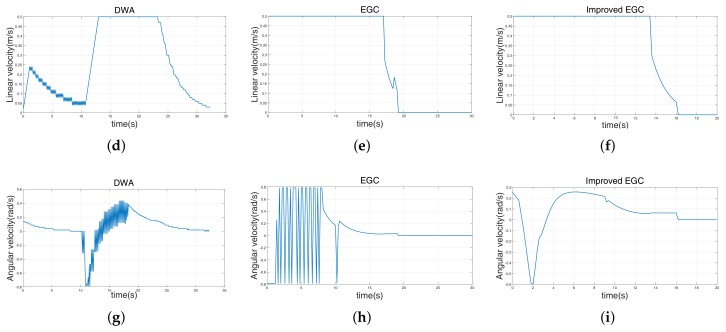
Simulation with a single obstacle using the DWA, EGC and improved EGC. (**a**–**c**) The trajectory of a robot in global coordinates. (**d**–**f**) Linear velocity. (**g**–**i**) Angular velocity.

As shown in Table 2, we compared the DWA, EGC, and IEGC with robot travel distance, time, and average algorithm running time. First, DWA responds sensitively to driving performance according to parameters. As mentioned above, we tried to find suitable parameters for parameter adjustment. The travel distance of the DWA is shorter than that of the EGC, but the total travel time is much longer. This is mainly because the obstacles and destinations from the robot are in a straight line, and the attempts to avoid the obstacle and to converge to the destination are inconsistent. As a result, the linear velocity is attenuated and the mobile robot can not avoid quickly. In addition, we can derive the optimum linear velocity and angular velocity considering the dynamics in the (v,w) space, but zig-zag motion still occurs, as shown in Figure 7g.

The EGC result is long compared to the DWA, but it takes less time to travel and has a shorter algorithm running time. In Figure 7h, a zig-zag motion appears near the obstacle. Finally, the proposed method has better performance compared to the DWA and EGC for obstacle avoidance performance in terms of travel distance and time. Figure 7c,f,i also shows that zig-zag motion does not appear and it reaches the destination quickly by smooth driving. Therefore, the environment with a single obstacle, the method proposed in this paper shows the best performance.

### 4.2. Simulation in the Environment with Multiple Obstacles

This experiment simulates the environment with multiple obstacles and simulates the same parameters as in the case of a single obstacle. We used the travel distance, time, and average algorithm running time as indexes for the algorithm evaluation.

**Figure 8:**
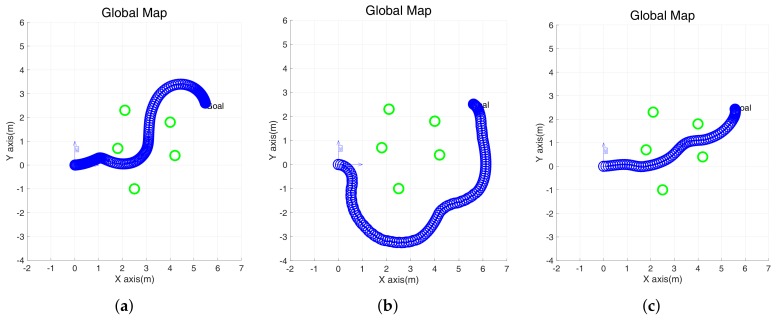

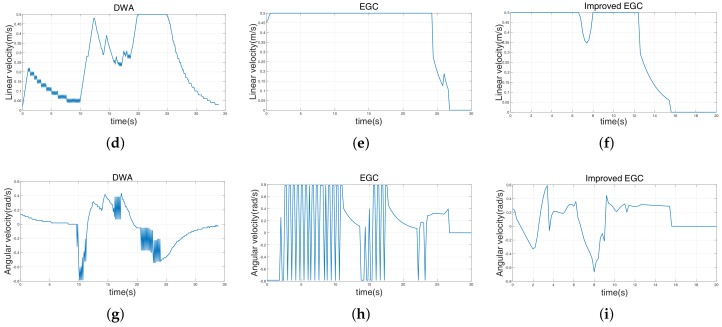
Simulation with multiple obstacles using the DWA, EGC, and improved EGC. (**a**–**c**) The trajectory of a robot in global coordinates. (**d**–**f**) Linear velocity. (**g**–**i**) Angular velocity.

Unlike in the environment with a single obstacle, the EGC, which is mainly based on bypassing, failed to enter between obstacles and bypassed the obstacle to reach the destination. As seen in Figure 8a, the DWA was also shown to bypass the narrow gap near the goal. However, as shown in Table 3, the proposed method passes through obstacles and reaches the destination with the shortest distance and time. Also, unlike the DWA and EGC in which zig-zag motion occurs, the proposed method smoothly runs between obstacles. It will be shown in the next section that the superior performance of the proposed method is valid in the real environment.

### 4.3. Experimental Results in a Real Environment with Multiple Obstacles

**Figure 9:**
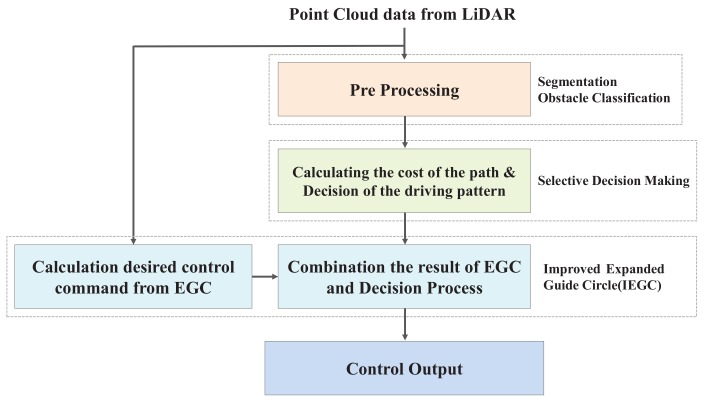
Structure of the improved Expanded Guide Circle with selective decision-making.

**Figure 10:**
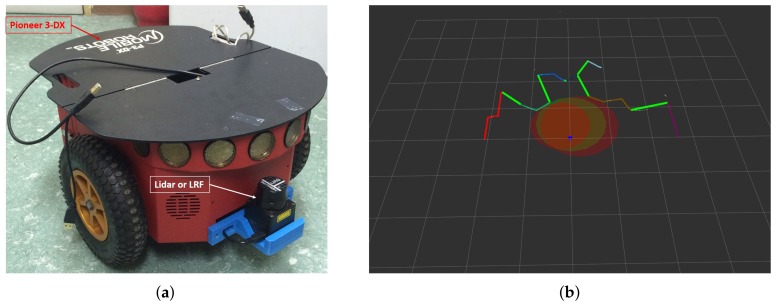
(**a**) Hardware Configuration. (**b**) Visualization the improved Expanded Guide Circle with selective decision-making using rviz in the Robot Operating System (ROS).

The hardware developed for the experiment is as follows. A differential-drive type mobile robot named Pioneer 3-DX (P3DX) from Omron Adept Tech in Pleasanton, California is used to demonstrate the performance of the proposed method in an indoor environment with obstacles. To interpret the kinematics of a robot, we need to know the width of the robot and the radius of the wheel. The width of the robot used in this experiment is 381 mm and the radius of the wheel is 97.5 mm. As shown in Figure 10a, a LiDAR sensor was attached to the front of the robot (URG-04LX-UG01 made by HOKUYO in in Osaka, Japan). The range of the LiDAR sensor is 20–5600 mm and $240^{\circ }$. The software is based on the Robot Operating System (ROS). The ROS is an open source system, providing a very large amount of information, so that it can easily provide a basis for experimenting with mobile robots. The algorithms implemented for testing in the real world work. In Figure 10b, the colored points are segmented laser scan points and the green lines are the feasible gaps. The red circle and green circle are the IGC and AGC described in Figure 3 and Figure 4. The method proposed in this paper is implemented according to the structure described in Figure 9. When point cloud data is first acquired from a LiDAR sensor, it is inserted as a pre-processing process for selective decision-making and the EGC algorithm, respectively. This control input is calculated from the linear and angular velocity of the robot and is given to the robot in Figure 10a. Finally, it is possible to drive safely to the final destination without a collision if the vehicle is driven in the real world by the proposed method in this manuscript.

In this experiment, three experiments were performed to verify the performance of the proposed method. At first, in the known environment, experiments were conducted for two cases. Experiments were conducted to show which of the two driving modes defined in Section 2.1 was more efficient.

**Figure 11:**
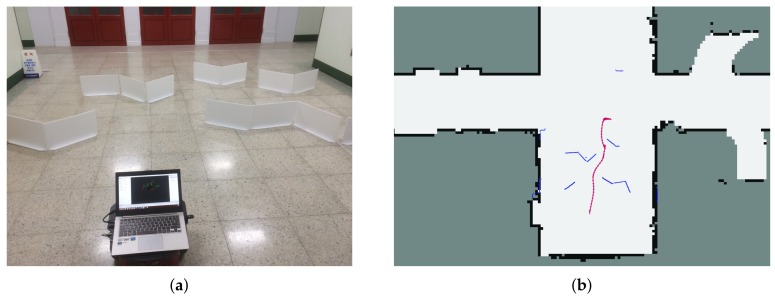

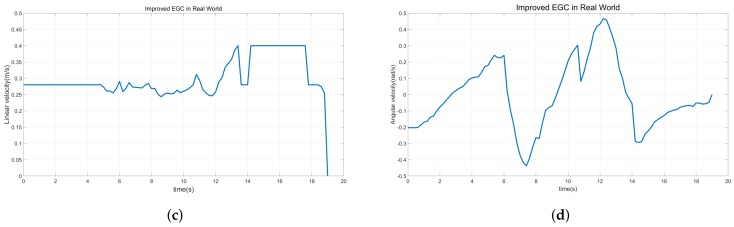
Experimental results in the real world. (**a**) The experimental environment. (**b**) The trajectory of the robot using the improved EGC with selective decision-making. (**c**) Linear velocity. (**d**) Angular velocity.

Figure 11b shows that it is more efficient to detour to the left side than to travel between obstacles. If the robot enters through obstacles, it has to pass through narrow passages and the speed of the robot is reduced when passing, which is inefficient in terms of total travel time. Therefore, the proposed method is able to reach the destination safely by selecting the bypass path. Also, as Figure 11 shows, the robot generates control commands to reach the destination quickly without a zig-zag motion at nearly full speed.

**Figure 12:**
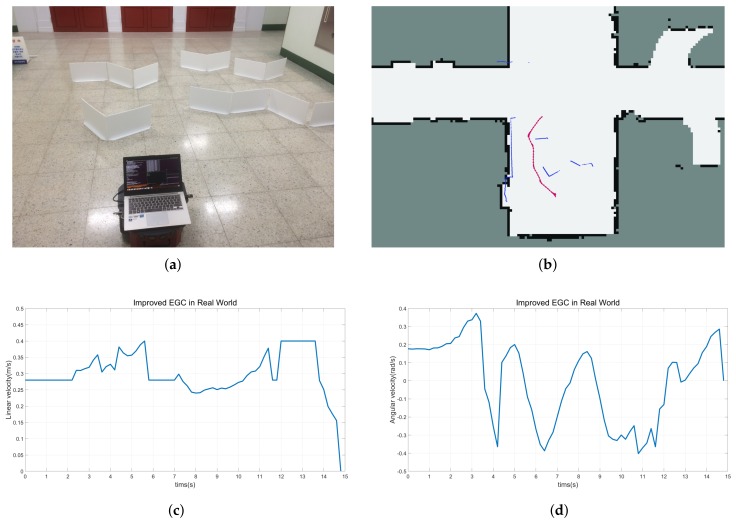
Experimental results in the real world. (**a**) Experimental environment. (**b**) The trajectory of the robot using the improved EGC with selective decision-making. (**c**) Linear velocity. (**d**) Angular velocity.

When the robot is unable to bypass obstacles or the Bypass mode is ineffective, the robot selects the Entry mode and passes the narrow passage safely to the destination, as shown in Figure 12. The scarlet arrows in Figure 11b and Figure 12b refer to the trajectory of the robot and blue points represent the surrounding environment of obstacles and walls.

**Figure 13:**
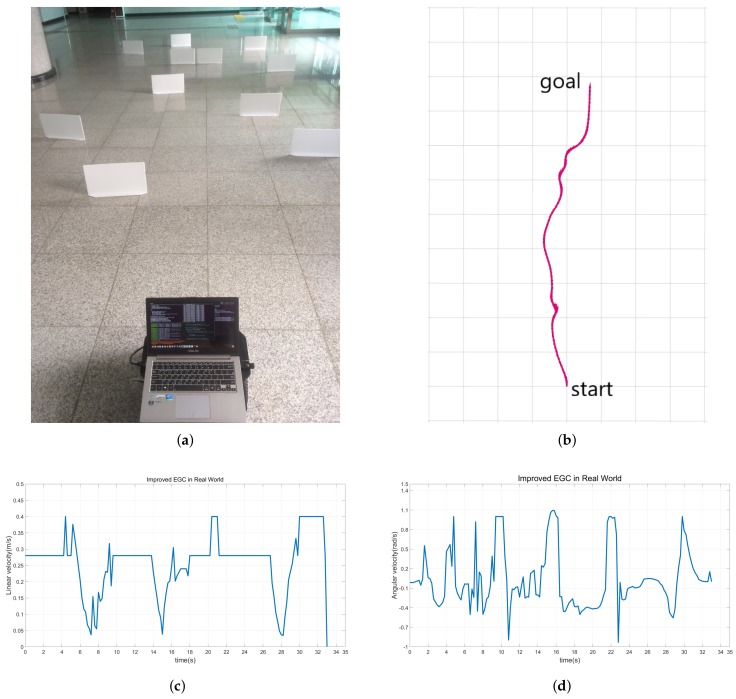
Experimental results in the real world. (**a**) The experimental environment. (**b**) The trajectory of the robot using the improved EGC with selective decision-making. (**c**) Linear velocity. (**d**) Angular velocity.

Finally, we validate the proposed method in an unknown environment. This experiment shows that the robot is driven safely to its destination in a situation where the map is not given and only an arbitrary destination is given. As shown in Figure 13a, obstacles are placed all over the place. Figure 13b shows the trajectory of the robot. This result shows that the destination is safely reached even though the map is not given.

## 5. Conclusions

In this paper issues pertaining to collision avoidance for the mobile robot are addressed. We have improved the Expanded Guide Circle and also have presented selective decision-making. Using the proposed method, smooth control is obtained without zig-zag motion while efficiently performing entering and bypassing operations. We also evaluated the validity by the simulations and experiments in a complex environment. The main contributions of the proposed method are the classifications of entry and bypass driving and the proposal of an efficient decision process to perform effective obstacle avoidance. The proposed method is applicable to various robot systems in general.

## Figures and Tables

**Table 1:** The obstacle avoidance algorithm using the improved Expanded Guide Circle.

**Algorithm** Obstacle Avoidance using the improved Expanded Guide Circle
**Input** Velocity commands toward goal ($v_{R}$, $w_{R}$), Scan data set $M=\{m_{i}\;:\;ith\;scan\;data\;on\;the\;point\;P_{m_{i}}=\left(x_{i},y_{i}\right)\;in\;the\;frame\;\left\{R\right\}\;for\;i=1,\cdots ,n\}$ and goal position $P_{goal}=\left(x_{goal},\;y_{goal}\right)$
**Output** Modified velocity commands ($v_{R}^{mod}$, $w_{R}^{mod}$) for avoiding the obstacles using the improved EGC
1: $r_{ur}\leftarrow$ the radius a few more than the width of the robot 2: $P_{LIGC}\leftarrow$ ${\left[r_{ur}\cdot \cos\left(w_{max}\right)\;r_{ur}\cdot \sin\left(w_{max}\right)\right]}^{T}$ 3: $P_{RIGC}\leftarrow$ ${\left[r_{ur}\cdot \cos\left(-w_{max}\right)\;r_{ur}\cdot \sin\left(-w_{max}\right)\right]}^{T}$ 4: $r_{LIGC}\leftarrow$ the shortest distance from $P_{LIGC}$ to the scan data $m_{i}$ in M 5: $r_{RIGC}\leftarrow$ the shortest distance from $P_{RIGC}$ to the scan data $m_{i}$ in M 6: 7: **if** $r_{LIGC}>r_{max}\;\&\;r_{RIGC}>r_{max}$ **then** 8: $r_{LIGC}\leftarrow r_{RIGC}\leftarrow r_{max}$ 9: $\left(v_{R}^{mod},w_{R}^{mod}\right)\leftarrow \left(v_{R},w_{R}\right)$ 10: **else** 11: $\theta_{AGC}\leftarrow weighted\;sum\;of\;\theta_{LIGC}\;and\;\theta_{RIGC}$ 12: $P_{AGC}\leftarrow$ ${\left[r_{ur}\cdot \cos\left(\theta_{AGC}\right)\;r_{ur}\cdot \sin\left(\theta_{AGC}\right)\right]}^{T}$ 13: $r_{AGC}\leftarrow$ the shortest distance from $P_{AGC}$ to the scan data $m_{i}$ in M 14: **if** $r_{AGC}>r_{max}$ **then** 15: $r_{AGC}\leftarrow r_{max}$ 16: **end if** 17: $P_{goal}\leftarrow Decision\;Process\left(M,P_{goal}\right)$ 18: $\theta_{goal}\leftarrow calculation\;of\;the\;angle\;from\;P_{goal}\;in\;the\;robot\;coordinate$ 19: $\theta_{IEGC}\leftarrow weighted\;sum\;of\;\theta_{AGC}\;and\;\theta_{goal}$ 20: $P_{IEGC}\leftarrow$ ${\left[r_{ur}\cdot \cos\left(\theta_{IEGC}\right)\;r_{ur}\cdot \sin\left(\theta_{IEGC}\right)\right]}^{T}$ 21: $r_{IEGC}\leftarrow$ the shortest distance from $P_{IEGC}$ to the scan data $m_{i}$ in M 22: **if** $r_{IEGC}>r_{max}$ **then** 23: $r_{IEGC}\leftarrow r_{max}$ 24: **end if** 25: ($v_{R}^{mod}$, $w_{R}^{mod}$) ← produce the modified operation command using $P_{IEGC}$ 26: **end if**

**Table 2:** The performance comparison of the Dynamic Window Approach (DWA), Expanded Guide Circle (EGC), and improved EGC (IEGC) algorithms in an environment with a single obstacle.

	DWA	EGC	IEGC
Distance (m)	8.478	8.954	7.204
Time (s)	32.3	19.2	16.2
Average Running Time (s)	0.0869	0.0011	0.0163

**Table 3:** A performance comparison of the DWA, EGC, and improved EGC algorithms in an environment with multiple obstacles.

	DWA	EGC	IEGC
Distance (m)	8.177	12.605	6.619
Time (s)	33.9	26.8	15.6
Average Running Time (s)	0.1957	0.0027	0.0214
